# Integrated multiomics approach identifies calcium and integrin-binding protein-2 as a novel gene for pulse wave velocity

**DOI:** 10.1097/HJH.0000000000000732

**Published:** 2015-09-25

**Authors:** Massimo Mangino, Marina Cecelja, Cristina Menni, Pei-Chien Tsai, Wei Yuan, Kerrin Small, Jordana Bell, Gary F. Mitchell, Phillip Chowienczyk, Tim D. Spector, AortaGen Consortium

**Affiliations:** a Department of Twin Research and Genetic Epidemiology, King's College London; b NIHR Biomedical Research Centre at Guy's and St. Thomas’ Foundation Trust; c Department of Clinical Pharmacology, King's College London; d The Institute of Cancer Research, London, UK; e Cardiovascular Engineering, Inc, Norwood, Massachusetts, USA

**Keywords:** arterial stiffness, arteriosclerosis, association, calcium and integrin-binding protein-2, pulse wave velocity, vascular calcification

## Abstract

**Background:**

Carotid-femoral pulse wave velocity (PWV) is an important measure of arterial stiffness, which is an independent predictor of cardiovascular morbidity and mortality. In this study, we used an integrated genetic, epigenetic and transcriptomics approach to uncover novel molecular mechanisms contributing to PWV.

**Methods and results:**

We measured PWV in 1505 healthy twins of European descendent. A genomewide association analysis was performed using standardized residual of the inverse of PWV. We identified one single-nucleotide polymorphism (rs7164338) in the calcium and integrin-binding protein-2 (*CIB2*) gene on chromosome 15q25.1 associated with PWV [β = −0.359, standard error (SE) = 0.07, *P* = 4.8 × 10^–8^]. The same variant was also associated with increased *CIB2* expression in leucocytes (β = 0.034, SE = 0.008, *P* = 4.95 × 10^–5^) and skin (β = 0.072, SE = 0.01, *P* = 2.35 × 10^–9^) and with hypomethylation of the gene promoter (β = −0.899, SE = 0.098, *P* = 3.63 × 10^–20^).

**Conclusion:**

Our data indicate that reduced methylation of the *CIB2* promoter in individuals carrying rs7164338 may lead to increased *CIB2* expression. Given that *CIB2* is thought to regulate intracellular calcium levels, an increase in protein levels may prevent the accumulation of serum calcium and phosphate, ultimately slowing down the process of vascular calcification. This study shows the power of integrating multiple omics to discover novel cardiovascular mechanisms.

## Abstract 2

Supplemental Digital Content is available in the text

## INTRODUCTION

Arterial stiffening represents a hallmark of vascular ageing. Carotid-femoral pulse wave velocity (PWV) is an important measure of central arterial stiffness [1]. A growing body of evidence supports the association between arterial stiffness and increased risk of developing ageing-related conditions such as myocardial infarction [2], hypertension [3], chronic kidney disease [4] and cognitive dysfunction [5]. Furthermore, PWV can be used as independent predictor of hypertension [6], coronary artery disease and stroke [7] in healthy individuals. Finally, a recent study suggests that molecular mechanisms related to arterial stiffening and cardiovascular mortality are not fully encompassed by the traditional cardiovascular risk factors [8]. To date, the exact cause of age-related aortic stiffening still remains unknown. Twin and family studies estimated that PWV has a heritability of approximately 40% [9–11]. Genomewide association (GWA) studies identified two main loci associated with PWV: collagen type 4 (*COL4A*), which is the major structural component of basement membranes [12], and the chromosome 14q32.2 locus that harbours a gene enhancer for the B-cell chronic lymphocytic leukaemia/lymphoma 11B (*BCL11B*) gene [10].

Our group previously reported that aortic stiffening is largely independent of classical risk factors for atherosclerosis [9]. We also demonstrated that, despite calcification often colocalizes with atherosclerotic plaque, arterial stiffness is associated with aortic calcification rather than coexistent atheromatous plaque [13]. Moreover, we showed that the link between PWV and calcification is mainly driven by genetic factors (heritability = 0.77) [13].

In this article, we used a systems-based approach combining genomics, transcriptomics and epigenomics, to identify novel molecular mechanisms contributing to PWV.

## METHODS

### Participants

The TwinsUK cohort (www.twinsuk.ac.uk, also referred to as the UK Adult Twin Register) is an adult twin British registry shown to be representative of the UK female population [14,15]. From this registry, a total of 1505 individuals had PWV measurements and were included in the analysis. The study was approved by the Research Ethics Committee of St. Thomas’ Hospital, London, UK, and all study participants provided informed written consent.

### Pulse wave velocity measurements

Vascular measurements were performed in a quiet temperature-controlled (22–24°C) vascular laboratory after at least 10-min rest. Brachial blood pressure was measured according to British Hypertension recommendations using a validated automated oscillometric device (Omron, 705 IT; Omron Healthcare, Kyoto, Japan) [16]. PWV was determined using the Sphygmocor system (Atcor, New South Wales, Australia) by sequentially recording carotid and femoral pressure waveforms using applanation tonometry, referenced to the R-wave of the electrocardiogram over a 10-s period. PWV was calculated as from path length/transit time. Transit time is measured using the intersecting tangent method and path length taken as the distance between the sternal notch and femoral artery at the point of applanation. Coefficient of variation between consecutive measures of PWV using the intersecting tangents method is less than 7% [17].

### Genotype

TwinsUK samples were typed with the Infinium 317K and 610K assay (Illumina, San Diego, California, USA; http://www.illumina.com/) at two different centres, the Centre for Inherited Diseases Research (USA) and the Wellcome Trust Sanger Institute. We pooled the normalized intensity data and called genotypes on the basis of the Illluminus algorithm. No calls were assigned if the most likely call was less than a posterior probability of 0.95. Validation of pooling was done by visual inspection of 100 random, shared single-nucleotide polymorphisms (SNPs) for overt batch effects; none were observed. We excluded SNPs that had a call rate less than 97% (for SNPs with MAF ≥ 5%) or less than 99% (for SNPs with 1% ≤ MAF < 5%), Hardy–Weinberg equilibrium *P* values less than 10^−6^ and minor allele frequencies less than 1%. We also removed individuals in whom the sample call rate was less than 98%; the heterozygosity across all SNPs was ≤ 2 standard deviations from the sample mean; there was evidence of non-European ancestry as assessed by principal component analysis comparison with HapMap3 populations; and the observed pairwise identity by descent probabilities suggested sample identity errors. Imputation of genotypes was carried out using the software IMPUTE V2 (https://mathgen.stats.ox.ac.uk/impute/impute_v2.html) [18]. Further quality controls (call rate ≥90%, MAF ≥0.01, Hardy–Weinberg equilibrium ≥10^–4^) were applied to the results post-GWA analysis.

### Genomewide association analysis

The GWA analysis was performed using standardized residuals. Those were obtained for the inverse of PWV adjusting for age, sex and BMI. As a result of the transformation, PWV had a normal distribution (mean of 0 and standard deviation of 1) across TwinsUK.

To account for family structure in the TwinsUK cohort, we utilized the GenABEL software package (http://www.genabel.org/) [19] which is designed for GWA analysis of family-based data by incorporating a pairwise kinship matrix calculated using genotyping data in the polygenic model to correct relatedness and hidden population stratification. The linear regression implemented in the software was used to test the association between a given SNP and PWV.

To validate our result, we obtained access to rs7164338 summary results generated by the AortaGen consortium as part of their study [10]. The nine populations included in AortaGen were of different origins with a 50 : 50 sex ratio [10]. A description of the populations included in the AortaGen study, as well as the statistical methods employed in their meta-analysis, has been reported in detail in Mitchell *et al.*[10].

The meta-analysis of TwinsUK and AortaGen results was performed using Han and Eskin's random-effect inverse variance method as implemented in the software METASOFT (http://genetics.cs.ucla.edu/meta) [20]. The Han and Eskin's methods have been shown to provide more robust results in the presence of heterogeneity [20]. In addition, to test the presence and measure the amount of between-study heterogeneity, we used two different metrics: Cochran's *Q* statistic [21] and *I*^2^[22]. A *P* value less than 0.05 in Cochran's *Q* test and *I*^2^ above 50% were considered evidences of large heterogeneity.

### Bioinformatic analysis

To query the publicly available data of the Encyclopedia of DNA Elements (ENCODE) project [23], we used HaploReg version 2 (http://www.broadinstitute.org/mammals/haploreg/haploreg.php) [24] to search for SNPs with functional annotations in high linkage disequilibrium (*r*^2^ > 0.8) with rs7164338 and RegulomeDB (http://regulomedb.org/) [25] to rank potential functional roles for SNPs identified by HaploReg. In the HaploReg analysis, we used the European population included in Phase 1 of the 1000 genome project for linkage disequilibrium calculation, the ENCODE data as source for epigenomes and both Genomic Evolutionary Rate Profiling (GERP) and SiPhy-omega algorithms to analyse the conservation in mammals.

The scoring system for RegulomeDB ranges from 1 to 6 with the strongest evidence for functional roles as scores 1 (a–f). A score of 2 includes evidence of transcription factor-binding and DNase footprint signals. Scores of 1 require additional evidence of effects of the SNP on specific gene expression.

### Expression analysis

We used the genomewide expression data from the lymphoblastoid cell lines (LCLs) and from the skin samples extracted from the Multiple Tissue Human Expression Resource (MuTHER) (www.muther.ac.uk) [26]. The project, established by the Wellcome Trust in 2007, provides a resource of genetic and genomic data from three tissues (lymphocytes, skin biopsies and subcutaneous fat). Gene expression was analysed with the Illumina Human HT-12 V3 chip [26]. The results generated by the project in the different tissue are publically available (http://www.muther.ac.uk/Data.html). MUTHER expression analyses were performed using GenABEL adjusting for the appropriate covariate (age, family structure and tissue batch) [26]. For this article, we extracted expression levels of calcium and integrin-binding protein-2 (CIB2) in LCL and Skin and rs7164338 from the online database. To validate our results in a different dataset, we used the online tool Genevar (V3.3.0) (https://www.sanger.ac.uk/resources/software/genevar/) [27]. Genevar is a Java application designed for the analysis and visualization of SNP–gene associations in different expression quantitative trait loci (eQTL) studies. We employed the option ‘cis-eQTL-SNP’ selecting the data generated by Stranger *et al.*[28] as reference study and testing a linear regression between the SNP and the gene after 10 000 nonparametric permutations.

Finally, tissue-specific (artery aorta) SNP-expression associations analysis was performed using data from the genotype-tissue expression (GTEx) project online portal (http://www.gtexportal.org) [29]. GTEx presents a comprehensive atlas of gene expression and regulation across multiple human tissues. The online database provides easy access to eQTLs, alternative splicing and the tissue specificity of gene regulatory mechanisms.

### Methylation analysis

DNA methylation levels for 69 probes spanned in a region 500K across CIB2 locus were obtained using the Illumina Infinium 450k chip array in 350 randomly selected individuals from the TwinsUK cohort. Similarly to the GWA and expression analyses, we tested the association between whole-blood DNA methylation patterns and rs7164338 using GenABEL [19]. The analysis was performed using residual of the methylation probes adjusted for age, sex and known source of batch effect (methylation chip, sample position on methylation chip and blood cell count).

To control for multiple testing, we calculated the effective number of tests from the correlation matrix for each methylation probe (69 × 69) using the method of Gao *et al.*[30]. After the pairwise correlation of the 69 analysed probes, the threshold of statistical significance was estimated at *P* less than 8.77 × 10^–4^.

The replication sample set included 172 individuals from TwinsUK. These samples were not related/overlapping with the discovery dataset. They were analysed using the Illumina HumanMethylation27 DNA Analysis BeadChip assay. As per the discovery, the validation analysis was performed using GenABEL [19] to correct for family structure and zygosity on the residuals of the methylation probes adjusted for age and sex, and know sources of batch effect (methylation chip, sample position on methylation chip and blood cell count). For the replication analysis, a *P* value less than 0.05 was considered significant as only one probe was tested.

### Mendelian randomization

We reported that methylation levels in the promoter region of *CIB2* (probe cg20761322) were associated with expression levels of the gene in LCLs. However, this observation cannot generally distinguish between association and causation [31]. In order to make causal inferences, we used a method called Mendelian randomization. This technique relies on the fact that genotypes are essentially random assortment of alleles at the time of gamete production and fertilization as indicated by Mendel's Second Law [31]. Hence, using a genetic variant (rs7164338) enables us to obtain unbiased estimates of the effects of a putative causal variable (cg20761322) without conducting a traditional randomized trial [32].

In Mendelian randomization analysis, we included all the samples (*n* = 221) that had data across three ‘omics’ (genetics, expression, methylation). We used the generalized method of moments with cluster-robust heteroskedastic-consistent variance estimates [33]. Under, weak and overidentification limitations were checked using *ivreg2* command in Stata (version 14; StataCorp LP, College Station, Texas, USA).

## RESULTS

The detailed characteristics of the participants included in this study are reported in Table 1. In brief, 1505 healthy twins with PWV measures available were included in the GWA analysis. They were of European descendent, mostly females (99.2%), with an average age of 59 years (±9 years). Their mean PWV was 9.3 m/s (±1.9 m/s) and their mean pulse pressure was 53.6 mmHg (±13.3 mmHg). The majority of the participants included in the analysis (80%) were not under antihypertensive medications.

We calculated that our study (*n* = 1505) had 80% power to detect a variant (minor allele frequency 10%) which has an effect on PWV of ±0.73 m/s at a statistical threshold of *P* ≤5 × 10^–8^ (Supplemental Figure 1).

**FIGURE 1:**
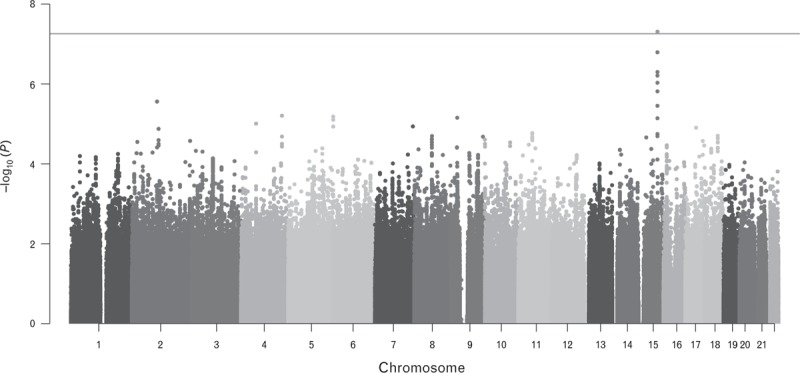
Manhattan plot of the genomewide association results. For each tested marker, the significance is displayed on the *y*-axis as the −log_10_ of the *P* value. The −log_10_ results are ordered along the *x*-axis by chromosome, with each coloured bar representing a different chromosome. The red line represents the genomewide significance threshold (5 × 10^–8^). In green is reported the lead single-nucleotide polymorphism rs7164338.

We performed a GWA to identify the genetic variations accounting for the inherited component of PWV. The result of the analysis is summarized in Fig. 1. The GWAS inflation (λGC) was 1.011, indicating that there was no significant population stratification or it was very minor. The quantile–quantile plot shows very little digression from the expected distribution under the null hypothesis (only present in the extremely low *P* values) (Supplemental Figure 2) confirming the absence of hidden relatedness and/or potential population stratification.

We, first, sought to replicate the two loci previously reported on chromosomes 13q34 (rs3742207) [12] and 14q32.2 (rs7152623) [10]. The power calculations showed that our sample size was not able to detect association for these two loci (rs3742207: 61%; rs7152623: 28%) at statistical significant level (*P* ≥ 0.01). Despite the association results of the two SNPs that were indeed not statistically significant (rs3742207: β = 0.08 ± 0.07, *P* = 0.31; rs7152623: β = −0.01 ± 0.04, *P* = 0.7), we observed that the effect sizes of the minor alleles were in the same direction in both cases. Altogether these results suggest that we may have replicated the previous findings but the sample size of our dataset affected the power to detect association for these two loci.

**FIGURE 2:**
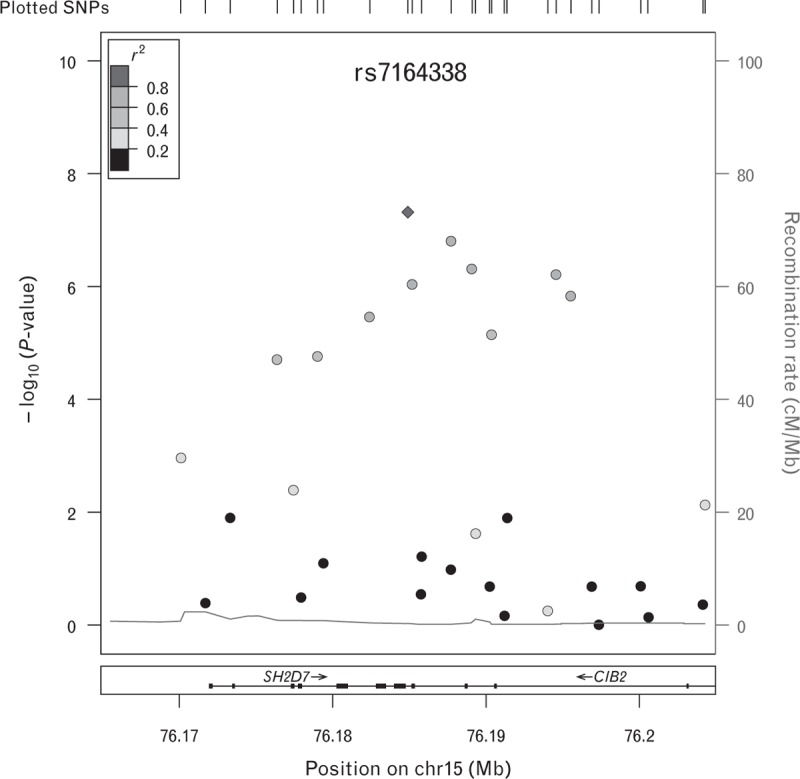
Regional plot of *CIB2* locus. Observed *P* values (−log_10_) are plotted against base-pair position. The lead SNP (rs7164338) is represented as a purple diamond and the linkage disequilibrium relationship (*r*^2^) with other SNPs in the region are indicated by the colour of the circles. Blue peaks represent recombination rates (HapMap 2), and the *RefSeq* genes are provided at the bottom. CIB2, calcium and integrin-binding protein-2; SNP, single-nucleotide polymorphism.

We identified 11 novel common variants associated with PWV in the *CIB2* gene on the long arm of chromosome 15 (15q25.1) (Fig. 2 and Table 2). The strongest association with PWV was observed for the intronic marker rs7164338 with a genomewide statistical significant *P* value equal to 4.8 × 10^–8^. To perform a technical validation, we compared rs7164338-imputed genotypes (rs7164338 had quality imputation score = 0.966) with the direct genotypes obtained from the next-generation sequence available for 49% of the samples included in the GWA. We observed 100% concordance between the next-generation sequence and the imputed genotypes. This may suggest that the association is not because of a technical artefact (i.e. imputation miscall). Furthermore, we tried to validate rs7164338 results in a different cohort. In particular, we request access to rs7164338 summary results generated by the AortaGen consortia [10]. Although this polymorphism was not significant in the AortaGen meta-analysis (*P* = 0.409) (Table 3), the effect of the minor allele (C) was in the same direction (β = −0.010 ± 0.012) (Table 3). The meta-analysis between the two datasets showed a suggestive *P* value of 5.87 × 10^–5^ (β = −0.177 ± 0.174) (Table 3). However, both *I*^2^ and Cochran's *Q* metrics detected a very high between-study heterogeneity (*I*^2^ = 95% and Cochran's *Q P* = 1.83 × 10^–6^) reflecting the distinct different demographics of our sample, which mainly included middle-aged women. Despite Han and Eskin's meta-analysis method that provides robust results in presence of heterogeneity [20], the results did not provide a conclusive evidence either to validate or reject the novel locus.

We, then, performed a conditional analysis using TwinsUK dataset, including rs7164338 as a covariate, to identify potential independent secondary signals at this locus. The results of this analysis (Supplemental Figure 3) did not find any significant evidence for an independent signal. Therefore, we looked for common variants (MAF ≥ 10% based on the European samples included in the 1000 Genomes Project) in tight linkage disequilibrium (*r*^2^ > 0.8) with the top SNP rs7164338 in order to identify potential causal alleles in the coding sequence. We identified nine SNPs (Supplemental Table 1) of which only one (rs10456, *r*^2^ = 0.86) was in *CIB2* coding region causing a synonymous change (aspartic acid to aspartic acid) in four over nine transcripts. However, the functional annotation analysis using data from the ENCODE [23] project on this polymorphism did not suggest any significant evidence for a potential functional role (Supplemental Table 1). Conversely, rs7164338 functional annotation analysis showed that this variation is located in an area of histone protein H3K4me1 chromatin modification associated with transcription enhancer and promoter sequences; altered regulatory motifs and affected one binding site for the transcription factor neuron-restrictive silencer factor (NRSF) (Supplemental Table 1). Altogether these evidences suggest that rs7164338 may have a potential regulatory function.

In order to explore rs7164338 theoretical functional impact, we analysed *CIB2* expression data from the MuTHER [26] (http://www.muther.ac.uk/) based on 856 unselected twins sampled for skin, adipose tissue and LCLs. We first focused our analysis on LCL and found that the minor allele (C) of rs7164338 was associated with higher expression of *CIB2* (ILMN_1714489, *P* = 4.95 × 10^–5^) (Table 4). These results were validated (*P* = 5.9 × 10^–3^) by analyzing LCL expression levels measured in 109 Centre d’Etude du Polymorphisme Humain (CEPH) individuals by Stranger *et al.*[28]. Owing to both Stranger *et al.* and MuTHER consortium utilized the same probe (ILMN_1714489) to assess the expression levels in the analysed tissues, we checked the presence of any genetic variant in the probe sequence which may have affected the efficiency of the hybridization and, consequently, our results. The analysis revealed that no polymorphism was present in the probe sequence.

Given the histological similarities between the central arteries and the skin (both are elastic connective tissues enriched by elastic fibres such as elastin [34]), we hypothesized that the analysis of the expressions level of *CIB2* in skin would give a more comparable result with its expression in the vascular tissue. Indeed, our results showed a stronger association (*P* = 2.35 × 10^–9^) of rs7164338 minor allele with *CIB2* expression levels in skin when compared with the LCL results (Table 4). Finally, we used the GTEx Portal [29] to examine the association rs7164338–*CIB2* specifically in the artery aorta tissue. Despite the small sample size included in the database for this tissue (*n* = 72), also in this case, the analysis highlighted a highly significant association (*P* = 4 × 10^–4^) between rs7164338 and *CIB2* expression levels.

We hypothesized that the different level of expression in the participant carrying the minor allele may be due to a methylation change. We tested the association between rs7164338 and the DNA methylation profile of 69 probes mapping across the *CIB2* locus. After correction for multiple testing, rs7164338 minor allele was significantly associated with lower methylation levels of two probes (cg20761322, *P* = 3.63 × 10^–20^, and cg20509675, *P* = 2.28 × 10^–11^) (Table 5).

The association between rs7164338 and cg20761322 was validated in further 172 independent samples (analysed with HumanMethylation27 DNA Analysis BeadChip assay) obtaining a similar highly significant association result (*P* = 3 × 10^–9^) (Table 5).

The association between rs7164338 and cg20761322 was of particular interest because this probe maps 6 bp upstream CIB2 start codon of (cg20509675 maps 3 bp downstream the start codon) suggesting a hypothetical regulatory effect on CIB2 expression. Therefore, using all TwinsUK individuals (*n* = 221) with both expression and methylation information available, we tested the relationship between expression and the methylation levels detecting a statistically significant association (β = −0.17 ± 0.07, *P* = 1.2 × 10^–2^) (Table 6) between cg20761322 and LCL *CIB2* expression levels. This observation, however, cannot distinguish between association and causation [31]. Therefore, to formally test the causal relationship between DNA methylation (probe cg20761322) in this region and expression of LCL *CIB2*, we performed a Mendelian randomization analysis utilizing rs7164338 as the instrumental variable [32]. Our results (based on the 221 samples with genomic/methylation/expression data) showed that cg20761322 may have a significant genotype-dependent causal effect on it (*P* = 6 × 10^–4^) (Table 6).

## DISCUSSION

In this article, we conducted a GWA analysis of PWV. The overall power calculations based on our dataset showed that we have 80% power to detect a genetic variant which has an effect on PWV of ±0.73 m/s at genomewide statistical level. Indeed, based on our current knowledge on common complex traits, this very large effect size is unlikely to be determined by a single SNP [35].

We identified a novel variant (rs7164338) on chromosome 15q25.1 in the *CIB2* associated with lower PWV. This finding was supported using a whole ‘omics’ approach. Some caution should be exercised in extending these results to other populations and further studies are needed to validate these results in independent cohorts matching our study characteristics (our study was mainly composed of females (99.2%) of European descendent). Recent studies reported a significant difference of arterial stiffness between women and men [36] and among different ethnic groups [37,38]. Indeed, although the minor allele effect was in the same direction, we were not able to fully validate our result in a dataset including nine different populations with a nearly 50 : 50 sex ratio [10]. Nevertheless, the findings reported here were supported by three independent ‘omics’ datasets performed with different techniques in different centres. Moreover, highly stringent threshold values have been applied for each analysis performed to minimize the possibility that our results are not true-positive findings.

CIB2 is part of the CIB1-related proteins family that are characterized by an EF-hand domain [39]. These proteins are activated to respond to intracellular levels of calcium (Ca^2+^) that play a pivotal role in Ca^2+^ intracellular homeostasis [39]. Moreover, numerous studies have implicated the paralogue CIB1 (38% identical and 59% similar to CIB2 [39]), in cardiac hypertrophy [40] and atrial fibrillation, and in valvular heart disease [41]. In particular, CIB1 is a master regulator of the calcineurin-nuclear factor of activated T cells signalling pathway [40]. Of note, this pathway has recently been implicated in vascular calcification via differentiation of vascular smooth muscle cells towards an osteoblast-like phenotype [42]. Considering the similarity with CIB1, it is plausible to hypothesize a functional role of CIB2 in arterial calcification via regulation of the calcineurin-nuclear factor of activated T cells pathway.

Another likely function of CIB2, which is not mutually exclusive, is the regulation of Ca^2+^ serum levels [43]. In particular, a number of epidemiological and experimental studies have implicated elevated level of serum Ca^2+^ in the initial stages of vascular calcification [44–47].

**FIGURE 3:**
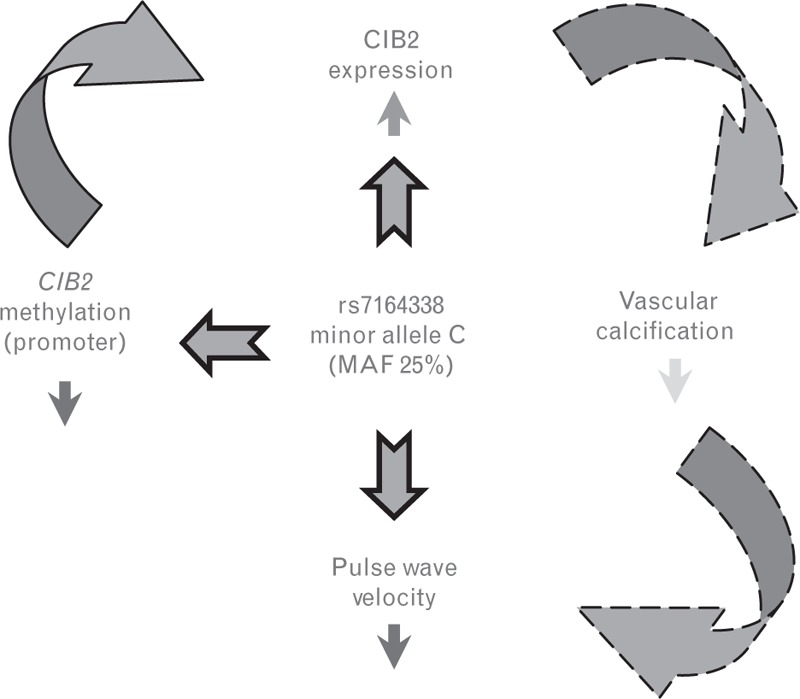
Effect of rs7164338 minor allele (C) on three analysed ‘omics’. We observed that rs7164338 minor allele was significantly associated (dark blue arrows) with lower (red arrow) methylation of *CIB2* promoter region and increased (green arrow) *CIB2* expression. We also reported a causal relationship between DNA methylation and *CIB2* expression. We hypothesized (dashed light blue arrows) that increased levels of *CIB2* expression in the individuals carrying the C allele may lead to increased arterial calcification (light blue arrow) and result in decrease (red arrow) of PWV observed in the GWA analysis. CIB2, calcium and integrin-binding protein-2; GWA, genome-wide association; PWV, pulse wave velocity.

We find that *CIB2* expression is mediated through a differentially methylated position in the promoter region. Therefore, we hypothesize a mechanism regulated by methylation of the *CIB2* promoter region in which *CIB2* may be more expressed in individuals carrying the rs7164338 minor allele resulting, as a consequence, in a less-accelerated vascular calcification and, ultimately, in a lower PWV (Fig. 3).

Our group have previously shown that the association of arterial stiffness with calcification is independent of coexistent atheromatous plaque [9]. The results reported in this article validate these observations [9,13], suggesting that arterial stiffness is the effect of arterial calcification and regulated by common genetic influence.

In conclusion, using a multi-‘omics’ approach, we provided the first evidences that *CIB2* may be responsible for PWV variation in humans, generating the foundation for future biological research in the calcium regulation and its connections with vascular ageing. This study also demonstrated new potential of a combined omics strategy in cardiovascular research.

## ACKNOWLEDGEMENTS

The authors are extremely grateful to all the twins who took part in this study, the midwives for recruiting them and the whole TwinsUK team, which includes interviewers, computer and laboratory technicians, clerical workers, research scientists, volunteers, managers, receptionists and nurses.

The study was funded by the Wellcome Trust; European Community's Seventh Framework Programme (FP7/2007–2013). The study also received support from the National Institute for Health Research (NIHR) BioResource Clinical Research Facility and Biomedical Research Centre based at Guy's and St Thomas’ NHS Foundation Trust and King's College London. SNP genotyping was performed by The Wellcome Trust Sanger Institute and National Eye Institute via NIH/CIDR.

### Conflicts of interest

G.F.M. is the owner of Cardiovascular Engineering, Inc, a company that designs and manufactures devices that measure vascular stiffness. The remaining authors report no conflicts of interest.

## Supplementary Material

SUPPLEMENTARY MATERIAL

## Figures and Tables

**TABLE 1:** Demographic characteristics of the study population (*n* = 1505)

Variable	
M:F	0.8%:99.2%
Age (years)	59.1 (±9.4)^a^
BMI (kg/m^2^)	26.5 (±4.9)^a^
PWV (m/s)	9.3 (±1.9)^a^
DBP (mmHg)	75.5 (±9.7)^a^
SBP (mmHg)	129.1 (±19.3)^a^
PP (mmHg)	53.6 (±13.3)^a^
Medication	
B-blockers	89 (5.9%)
Diuretics	125 (8.3%)
Calcium-channel antagonists	78 (5.2%)
ACE inhibitors	99 (6.6%)
Angiotensin receptor inhibitors	54 (3.6%)
No medication	1200 (80%)
Not known	11 (0.7%)

ACE, angiotensin-converting enzyme; PP, pulse pressure; PWV, pulse wave velocity.

^a^Mean (standard deviation).

**TABLE 2:** Summary results for the newly identified single-nucleotide polymorphisms associated with pulse wave velocity on chr 15q25.1

SNP	Chr	Position	Effect allele	Effect allele frequency	β (SE)	*P* value
rs11639461	15	76176384	C	0.23	−0.320 (0.075)	1.98 × 10^−05^
rs2867922	15	76179024	A	0.23	−0.320 (0.074)	1.75 × 10^−05^
rs9806257	15	76182417	C	0.30	−0.284 (0.066)	3.44 × 10^−06^
rs7164338	15	76184901	C	0.25	−0.359 (0.072)	4.80 × 10^−08^
rs10456	15	76185201	A	0.24	−0.330 (0.073)	9.13 × 10^−07^
rs11072728	15	76187728	A	0.25	−0.347 (0.071)	1.58 × 10^−07^
rs11072729	15	76189056	C	0.26	−0.323 (0.070)	4.92 × 10^−07^
rs2304829	15	76190347	C	0.28	−0.298 (0.069)	7.05 × 10^−06^
rs12440984	15	76194548	C	0.24	−0.336 (0.072)	6.02 × 10^−07^
rs8032449	15	76195510	A	0.29	−0.307 (0.067)	1.50 × 10^−06^
rs11630013	15	76221978	A	0.17	−0.324 (0.080)	7.56 × 10^−05^

SE, standard error; SNP, single-nucleotide polymorphism.

**TABLE 3:** Meta-analysis results for rs7164338^a^

Dataset	*N*	MAF	β	SE	*P* values	*I*^2^	Het *P*
TwinsUK	1505	0.25	−0.359	0.072	4.80 × 10^−08^		
AortaGen Consortium	20 634	0.25	−0.010	0.012	4.09 × 10^−1^		
Combined	22 139	0.25	−0.177	0.174	5.87 × 10^−5^	95%	1.83 × 10^–6^

Het *P*, heterogeneity *P*; MAF, minor allele frequency; SE, standard error.

^a^β and SE values refer to the minor allele C in both TwinsUK and AortaGen datasets.

**TABLE 4:** Association results for rs7164338 and calcium and integrin-binding protein-2 expression levels in lymphoblastoid cell lines (LCLs) and skin from the Multiple Tissue Human Expression Resource (MuTHER)^a^

Probe	β (SE)	*P* value
ILMN_1714489 (LCL)	0.034 (0.008)	4.95 × 10^−05^
ILMN_1714489 (Skin)	0.072 (0.012)	2.35 × 10^−09^

CIB2, calcium and integrin-binding protein-2; SE, standard error.

^a^β values refer to the effect allele C.

**TABLE 5:** Association summary statistic for rs7164338 and the methylation probes after correction for multiple testing in the discovery dataset^a^

Probe	Discovery		Replication	
β (SE)	*P* value	β (SE)	*P* value
cg20761322	−0.899 (0.098)	3.63 × 10^−20^	−0.022 (0.004)	3 × 10^–9^
cg20509675	−0.611 (0.091)	2.28 × 10^−11^	NA	NA

NA, not available; SE, standard error.

^a^In the replication sample, only cg20761322 was available for the analysis. β values refer to the effect allele C.

**TABLE 6:** Summary results of the Mendelian randomization

Locus	SNP	EA/OA	Methylation probe	*N*	Association (SNP–expression)		Association (SNP–methylation)		Association (methylation–expression)		Mendelian randomization (SNP → methylation → expression)	
β (SE)	*P* value	β (SE)	*P* value	β (SE)	*P* value	β (SE)	*P* value
CIB2	rs7164338	C/T	cg20761322	221	0.4 (0.11)	4.8 × 10^−04^	−0.84 (0.12)	8.3 × 10^−11^	−0.17 (0.07)	1.2 × 10^−02^	−0.48 (0.14)	6 × 10^−04^

CIB2, calcium and integrin-binding protein-2; EA, effect allele; OA, other allele; SE, standard error; SNP, single-nucleotide polymorphism.
