# Supplementary material for: Integrated multiomics approach identifies calcium and integrin-binding protein-2 as a novel gene for pulse wave velocity
Source: J Hypertens. 2015 Sep 25;34(1):79–87. doi: 10.1097/HJH.0000000000000732 (PMC4845763; doi:10.1097/HJH.0000000000000732)
Supplement: SUPPLEMENTARY MATERIAL [file jhype-34-79-s001.docx]

**Supplemental Material**

**Supplemental Table 1**. Functional regulation annotation for common SNPs in high LD (r2>0.8) with rs7164338 using HaploReg and RegulomeDB. The r^2^ values represent the LD between the relative marker and rs7164338.

| **rsID** | **Position (BUILD37)** | **r^2^** | **Ref/Alt. Allele** | **SNP Score*** | **Cons.^#^** | **Promoter histone marks** | **Enhancer**  **histone marks** | **Proteins bound** | **Motifs**  **changed** | **dbSNP**  **functional annotation** |
| --- | --- | --- | --- | --- | --- | --- | --- | --- | --- | --- |
| rs62009270 | 78394979 | 0.84 | G/C | 5 |  |  |  |  | KAP1 | intronic |
| rs62009271 | 78395196 | 1 | C/T | 5 |  |  | GM12878 |  | PU.1, p53 | intronic |
| rs9806257 | 78395362 | 0.84 | T/C | 5 |  |  | GM12878 |  | ELF1, Pax-6, SEF-1 | intronic |
| **rs7164338** | **78397846** | **1** | **T/C** | **1f** |  | **HepG2** | **GM12878, HSMM** | **NRSF** | **HEY1, Hic1, NRSF** | **intronic** |
| rs10456 | 78398146 | 0.86 | G/A | 4 | ++ | HepG2 | GM12878, HSMM, Huvec |  |  | synonymous |
| rs11072727 | 78400311 | 1 | C/T | NA |  |  |  |  | BDP1 | intronic |
| rs11072728 | 78400673 | 1 | T/A | 1b |  |  |  | CTCF | GCNF, Maf | intronic |
| rs59999498 | 78401335 | 1 | A/C | 5 |  |  |  |  | BDP1, PLAG1, PPAR, RREB-1, RREB-1, SREBP, TR4, VDR | intronic |
| rs11072729 | 78402001 | 0.97 | A/C | 6 |  |  |  |  | COMP1 | intronic |

* Regulome DB score: 1f =eQTL + TF binding / DNase peak; 1b= eQTL + TF binding + any motif + DNase Footprint + DNase peak; 4 TF binding + DNase peak; 5 TF binding or DNase peak; 6 other.

# conserved regions in mammals predicted by GERP and SiPhy


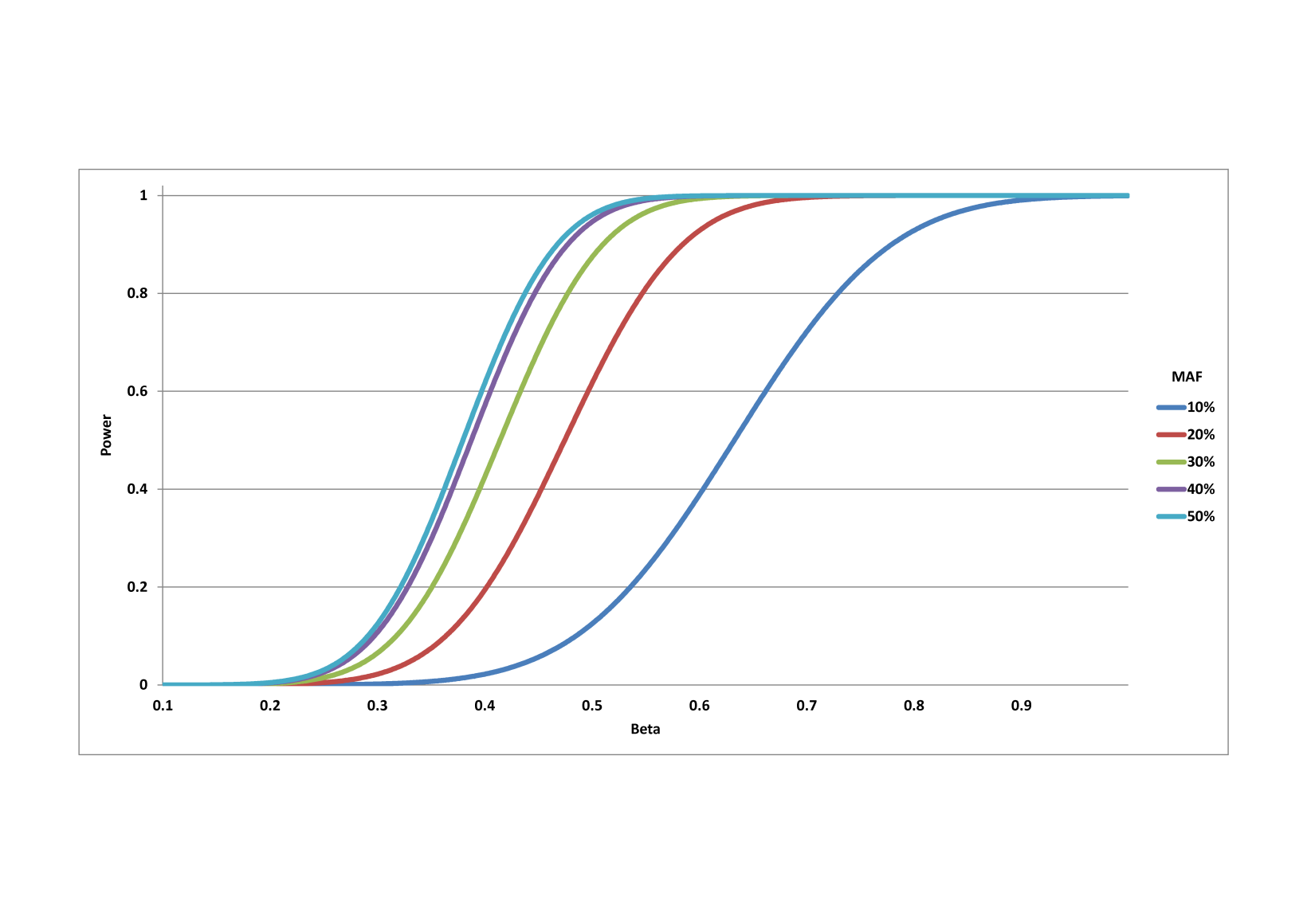


**Supplemental figure 1. Power calculation**. The figure represents the power (y-axis) of our cohort (n=1505) to detect effects of various sizes (beta, x-axis) at a genome-wide significance level (P=5x10^-8^) for variants with allele frequencies ranging from 10% (dark blue line, extreme right-hand curve) to 50% (light blue line, extreme left-hand curve).


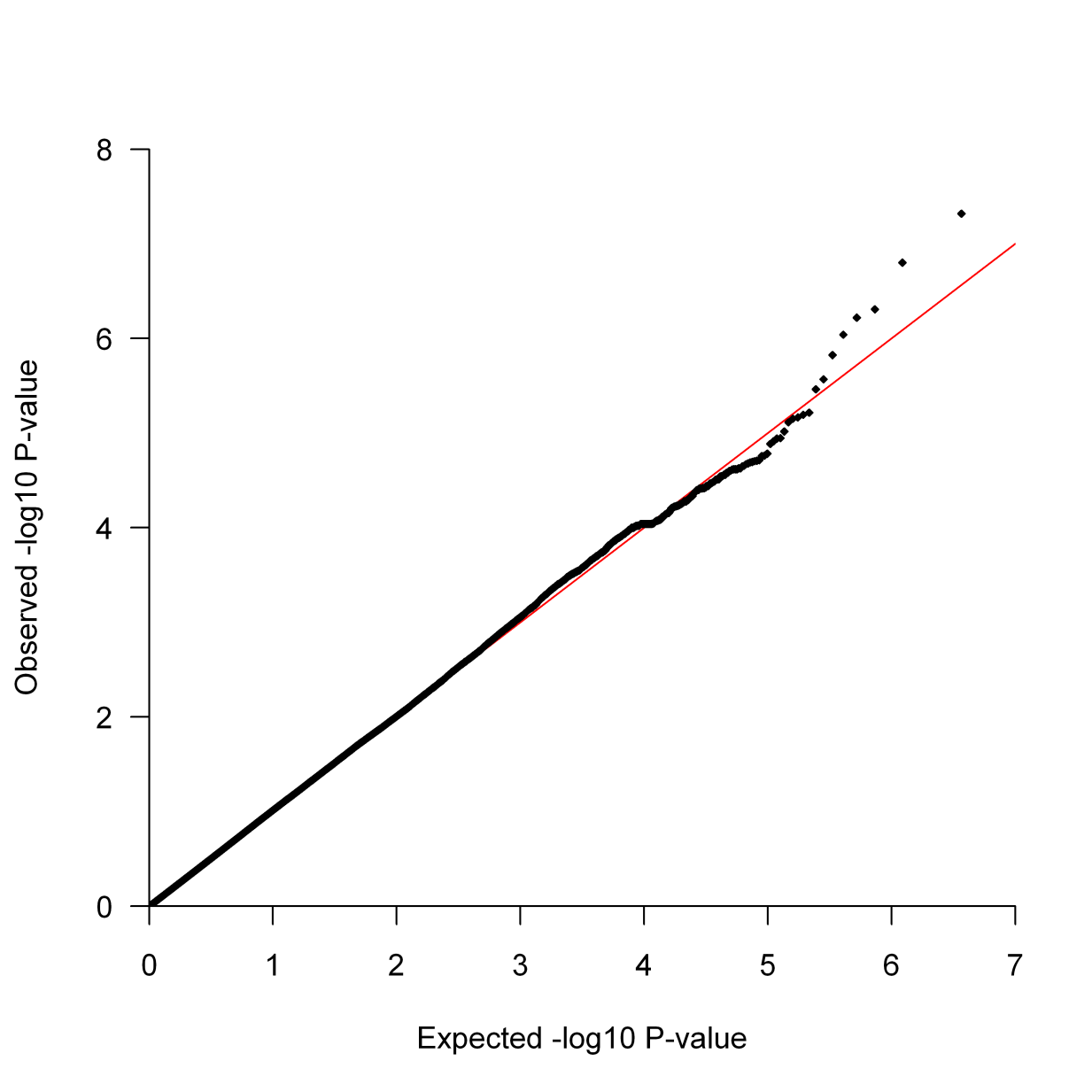


**Supplemental figure 2.** Quantile-quantile plot of observed P-values (black dots) versus expected P-values (red dots) plotted as –logP of the genome-wide association analysis of our cohort.


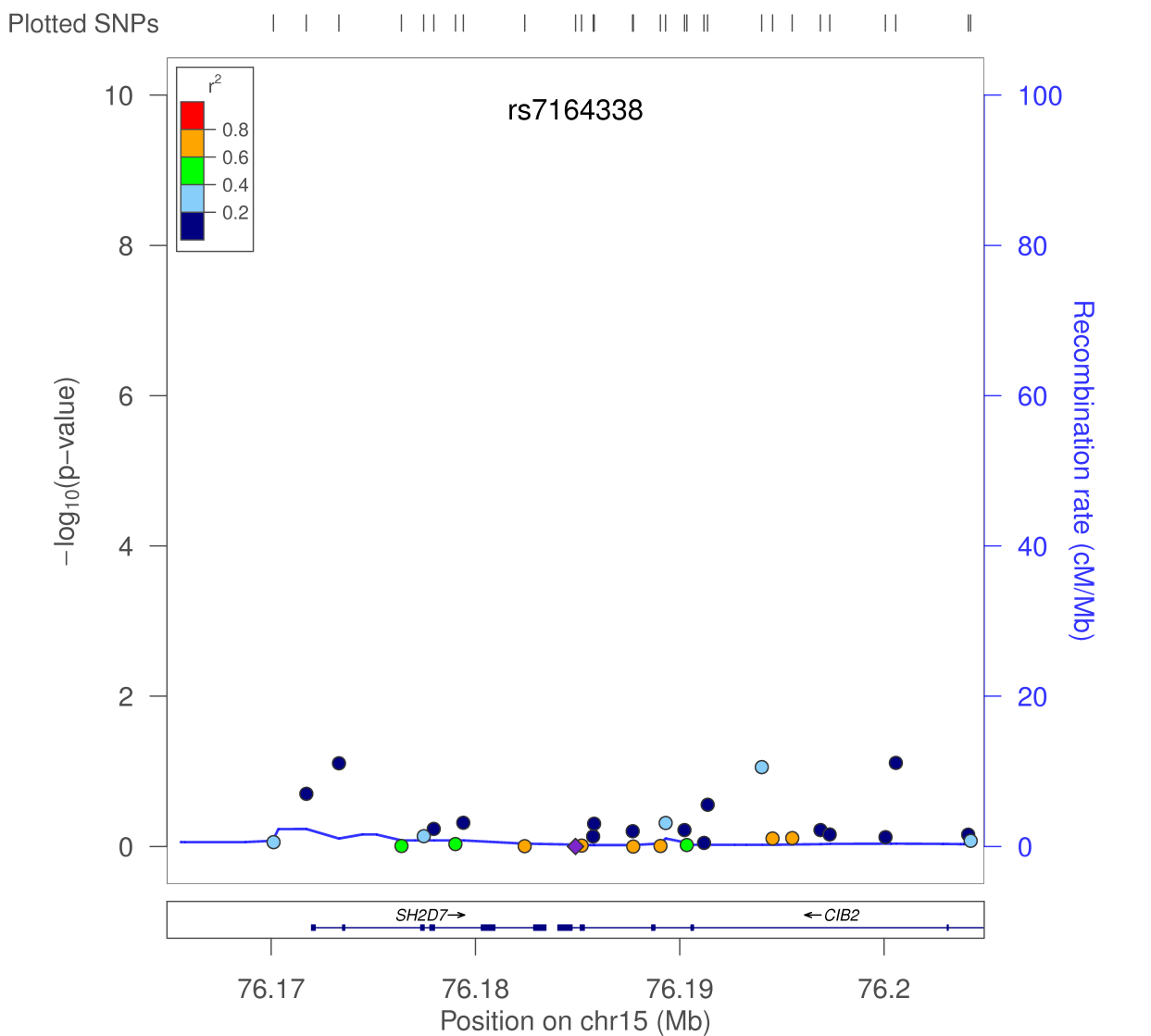


**Supplemental Figure 3.** **Regional plot of CIB2 locus after conditional analysis .** Observed Pvalues (-log10) are plotted against base-pair position. The association p-values of the lead SNP rs7164338 (represented as a purple diamond), as well as all the ones of the SNPs in linkage disequilibrium (r2),are not statistically significant after the conditional analysis, indicating the absence of any additional independent secondary signals at this locus. Blue peaks represent recombination rates (HapMap 2), and the RefSeq genes are provided at the bottom.
